# Tumor Promoters and Opportunities for Molecular Cancer Prevention

**DOI:** 10.1158/2159-8290.CD-24-0128

**Published:** 2024-07-01

**Authors:** William Hill, Clare E. Weeden, Charles Swanton

**Affiliations:** 1 Cancer Evolution and Genome Instability Laboratory, The Francis Crick Institute, London, UK; 2 Cancer Research UK Lung Cancer Centre of Excellence, University College London Cancer Institute, London, UK; 3 Department of Oncology, University College London Hospitals, London, UK

## Abstract

Environmental carcinogens increase cancer incidence via both mutagenic and non-mutagenic mechanisms. There are over 500 known or suspected carcinogens classified by the International Agency for Research on Cancer. Sequencing of both cancerous and histologically non-cancerous tissue has been instrumental in improving our understanding of how environmental carcinogens cause cancer. Understanding how and defining which environmental or lifestyle exposures drive cancer will support cancer prevention. Recent research is revisiting the mechanisms of early tumorigenesis, paving the way for an era of molecular cancer prevention.

## Cancer Initiation: Beyond the Genome

A longstanding model of carcinogenesis is that acquisition of mutations is the rate-limiting step toward tumor formation and that many environmental carcinogens act via mutagenesis. However, several lines of evidence, including seminal breakthroughs from the Mutographs Cancer Grand Challenges team, paint a more nuanced view of cancer initiation. (i) Somatic cells become replete with oncogenic mutations over time. Recent studies of sequencing histologically normal tissue from a range of tissue sites have identified oncogenic driver mutations without any evidence of disease, suggesting driver mutations are necessary but not always sufficient for tumorigenesis (1). (ii) Common oncogenic driver mutations may not be directly induced by cancer risk factors. Mutational signature analysis can be used to study the etiology and origins of mutations in the genome, but many oncogenic driver mutations do not arise within well-established environmental mutational signatures. For example, in silico analysis suggests common oncogenic drivers (KRASG12D, TP53R175H, and NRASQ61K) correspond within an aging-associated mutational signature (2). These observations challenge the notion that the mechanism of action for environmental carcinogens, such as UV light and tobacco smoking, are solely mutagenic (2). (iii) Many carcinogens drive cancer without altering mutational burden or leaving a clear mutational signature. A recent study by Balmain and colleagues of 20 International Agency for Research on Cancer group-one carcinogens found only three increased mutational burden and led to mutational signatures, whereas the majority accelerated cancer independently of mutagenesis (3). (iv) Mutational profiling alone may not fully explain geographical differences in esophageal cancer incidence. Profiling of over 500 whole genomes from esophageal tumors from eight different countries with varying esophageal cancer incidence rates revealed that the mutational profiles were strikingly similar (4). This lack of any clear mutational difference between tumors from high-risk and low-risk areas suggests that carcinogens that leave no measurable genomic imprint may promote cancer initiation. (v) In lung cancer arising in people who smoke (a clear mutagen), 8% of lung cancers lacked evidence of smoking-mediated carcinogenesis (5). This suggests that even upon exposure to a strong mutagen, tobacco smoke may accelerate cancer via alternative mechanisms (6). This is important as carcinogens are estimated to be responsible for around 40% of all cancer burden; however, some calculations suggest that up to 80% of the cancer burden is due to environmental or lifestyle exposures, both known and unknown (7). Altogether, these data highlight the additional impact of carcinogens beyond somatic mutagenesis, raising questions as to how many carcinogens drive cancer and how assays can be optimized to predict cancer risk associated with the introduction of new chemical matter into the human environment.

## Cancer Promotion

A two-step model of tumorigenesis was proposed in 1947 by Berenblum and Shubik. They postulated that skin carcinogenesis requires an initiation step, exposure to a mutagen, and a second step, exposure to an irritant inflammatory agent, the promotion step (8). Critically, without the promotion phase, mutant cells remain dormant in the skin for most of the lifespan of a mouse. Exposure to the promoter agent can be given 1 year after initiation of the mutation and still drive rapid tumorigenesis, indicating these latent cells harbor the capability of forming a tumor. Many environmental factors can be viewed through the lens of tumor promotion, the process that enables initiated cells to form tumors via multiple non-mutagenic mechanisms, including producing co-operating inflammation, generating susceptible cell lineages, increasing daughter cell production, allowing tissue colonization and escape from tissue architecture, and promoting immune evasion (9). Critically, a thorough understanding of the precise molecular mechanisms by which environmental carcinogens promote cancer may reveal potential intervention strategies.

## Examples of Tumor promotion

The lung is continually exposed throughout life by challenges such as bacteria, inhaled air pollutants, and tobacco smoking. Whilst tobacco smoking is a clear mutagen and tumor initiator, it also has been identified as a tumor promoter. Tobacco exposure leads to pulmonary inflammation characterized by elevated TNF-α and IL-6 levels and accelerates lung cancer in both genetic and carcinogen-driven models (6). Deletion of IKKβ within the myeloid compartment was sufficient to inhibit inflammation and malignant cell expansion. Asbestos is another environmental carcinogen which may act via a tumor promotion mechanism (10). Asbestos can cause high-mobility group box 1 (HMGB1) release from necrotic human mesothelial cells, triggering an inflammatory cascade involving IL-1β, TNF-α, and CXCL2. Finally, particulate matter air pollution may also promote lung adenocarcinoma via macrophage influx and IL-1β release (5). In line with the promoter model, oncogenic mutations in EGFR and KRAS have been detected in non-cancerous lung tissue.

Cancer risk factors are known to affect the development of pancreatic cancer. It has long been known that the adult mouse pancreas is refractory to transformation by oncogenic KRAS, despite the majority of patient pancreatic tumors harboring these mutations, yet the induction of experimental pancreatitis via caerulin exposure rapidly drives tumorigenesis (11). A high-fat diet can also activate oncogenic KRAS and COX-2 to promote cancer (12). Recent work has demonstrated an inflammatory loop between tumor-derived prostaglandin E2 (PGE2) and macrophage production of IL-1β to drive pathogenic inflammation and promote pancreatic ductal adenocarcinoma (PDAC; ref. 13). Upon pancreatic tissue injury, acinar cells shut off digestive enzyme production and de-differentiate to a progenitor-like state to facilitate regeneration after injury, a process termed acinar–ductal metaplasia. Activation of the PGE2–IL-1β axis could represent a physiological tissue injury response to maintain homeostasis which in the presence of an oncogenic driver mutation is diverted toward cancer. In line with this, inflammation can synergize with oncogenic mutations to trigger rapid chromatin remodeling within epithelial cells, establishing perturbed epithelia-immune communication feedback loops leading to elevated IL-33 expression that drives disease progression (14). In addition, analysis of adult pancreata from 30 donors spanning a wide range of ages and backgrounds found that 60% of healthy adults contain pancreatic epithelial neoplasia, precursor lesions to PDAC (15). The incidence of these lesions was unrelated to age, with lesions identified in individuals in their twenties. Given PDAC onset is typically decades later, this offers the opportunity for cancer interception and prevention. It is also clear that additional mutations, such as in p53, can accelerate pancreatic cancer in mouse models, although p53 mutations have pleiotropic effects on both the mutant cells and surrounding microenvironment ranging from metabolic rewiring, DNA damage response, modulation NF-κB inflammatory response, extracellular matrix remodeling, and mediating cell competition (16). Further work is required to understand the environmental and molecular triggers of progression of these preinvasive lesions to cancer and if targeting of these lesions reduces disease onset. Altogether, this highlights how latent mutant cells harboring oncogenic mutations can be triggered by external factors to expand and drive malignancy.

In addition to altering cell intrinsic signaling, oncogenic mutations in cancer cells alter the immune microenvironment in mouse models. For example, KRAS downstream pathways lead to the induction of IL-10, TGF-β, and GM-CSF within epithelial cells (17). Oncogenic mutations in mouse intestinal organoid co-cultures also disrupt fibroblast–epithelial communication, trapping epithelia in highly proliferative stem cell fates (18). It will be important to determine how mutant cell fate and cell–cell signaling is regulated by the combination of oncogenic mutation and environmental exposure.

## Epithelial Tumor Restraint and Promotion

Epithelial tissues have mechanisms to detect and eliminate cells harboring oncogenic mutations. Mutant cells can be outcompeted by adjacent normal neighbors in the intestine, pancreas, skin, lung, and esophagus (19–22). In this process, mutant cells are actively detected, typically by cell surface markers. For example, expression of oncogenic HRAS leads to autonomous upregulation of EphA2, which binds to ephrin receptors on adjacent normal cells, promoting mutant cell extrusion in vitro (23). Within the mutant cell, epithelial protein lost in neoplasm (EPLIN) accumulates and promotes filamin formation in normal neighboring cells. Inhibition of cell competition leads to accumulation of aberrant cells and is thought to promote preinvasive disease (24). Therefore, suppression of epithelial cell competition could be another mechanism of tumor promotion. In support of this, in mice fed a high-fat diet, extrusion of oncogenic Ras mutant cells is suppressed, leading to more tumor lesions in the pancreas (25). Mediators of inflammation can also alter extrusion, with PGE2 upregulated in in vitro co-cultures of normal and HRAS mutant cells with PGE2 inhibiting extrusion of mutant cells both in vitro and in mouse models of sporadic oncogenic HRAS expression (26). Further work is needed to determine if putative tumor-promoting agents can act via inhibition of cell competition causing accumulation of aberrant cells and early disease.

Epithelial tissues also balance the proliferation and differentiation of cells; one well-characterized example is the skin epidermis, in which progenitor cells are maintained at the basal layer and differentiate into keratinocytes that are eventually shed. Evidence suggests that when a cell divides, the adjacent neighbor is biased toward differentiation, known as local fate coordination (27). The ability to adhere to the basement membrane appears to be an important mediator of cell competition, with cells that have reduced COL17A1 able to be outcompeted by high-COL17A1 cells (28). Low-COL17A1 cells have decreased hemidesmosome attachments and as such are forced to divide perpendicularly, biasing daughter cell fate to differentiation. Over time, the differential bias in cell fate of COL17A1 low versus high cells leads to the elimination of less-fit COL17A1 cells. It has been found that COL17A1 is broken down during genomic and oxidative stress and as such there may be potential for environmental agents to alter COL17A1 levels and modulate competition in the skin. The tissue level differentiation bias can also be perturbed by genomic mutations. In the esophagus, cells harboring NOTCH1 mutations not only have a biased daughter cell fate toward generating progenitor cells but also promote the neighboring wild-type cells to differentiate and be lost (21). In another study exploring multi-organ cancer risk, stem and non-stem cell populations in mouse organs were tested for cancer susceptibility using oncogene and tumor-suppressor alleles and lineage tracing. It was found that life-long generative capacity, quantified as the difference in percentage of labeled cells between early and aged organs, is a key marker of risk of cancer development (29). This highlights how alterations in daughter cell fate can lead to clonal expansion or elimination and can be modulated by properties such as adherence to the niche and mutations.

The impact of environmental exposures on epithelial tissue homeostasis has been studied in non-cancerous contexts. For example, cigarette smoke decreases tight junctions (TJ) and adherens junctions, impacting adhesive intercellular junctions and leading to increased lung alveolar epithelial permeability associated with chronic obstructive pulmonary disorder (30). Air pollutants such as diesel exhaust and fine particulate matter damage components of TJ, such as claudin-1 and Zo-1, and suppress levels of E-cadherin in mouse models. Other exposures can also alter epithelial biology; for example, even at trace concentrations, cleaning detergents can disrupt TJ integrity in human keratinocytes and lung bronchial epithelium in vitro (30). The impact of environmental stresses on stem cell fate has also been explored (31). Mice exposed to tobacco smoke for 12 weeks identified increased Oct4 and Nanog expression within liver cells (32). Long-term in vitro exposure of immortalized human bronchial epithelial cells to cigarette smoke extract for 25 weeks induced expression of CD133 and ALDH1 and functionally increased organoid-forming efficiency via the Wnt/β-catenin pathway (33). In support of this, lung basal cells isolated from smokers show increased organoid-forming efficiency correlated with years of tobacco smoking (34). Exposure of human bronchial epithelial cells to cigarette smoke condensate for 15 months induces chromatin changes, leading to abnormal DNA methylation and anchorage-independent growth (35). Finally, inflammation and the associated reactive oxidative stress can modify G-rich telomere sequences, leading to uncapping, accompanying breakage–fusion–bridge cycles, and chromosome instability (36). Telomere dysfunction also activates YAP1 and upregulation of IL-18, leading to recruitment of IFN-γ-secreting T cells and tissue inflammation (37). Thus, the mechanisms by which exposures alter epithelial tissue biology, such as cell–cell adhesion, telomere dysfunction, and stem-like characteristics, have been identified; however, how these environmental exposures and biological changes promote cancer requires further elucidation.

In addition to host epithelia and microenvironment, our bodies are colonized by microorganisms that can modulate cancer promotion [reviewed in ref. 38]. The microbiome can directly interact with host cells and produce metabolites that trigger inflammation. Research has focused on bacteria, but fungi and viruses also may play roles in tumorigenesis. Bacteria such as Helicobacter pylori can activate NF-κB signaling and inflammation via components of the cell membrane or proteins it injects into cells, which can promote cancer (39). Another example is PDAC, where oncogenic Kras upregulates IL-33 but secretion is dependent on the intratumoral fungal mycobiome (40). The microbiome and mycobiome offers attractive targets for cancer prevention with opportunities for modulation via diet variations or medication. Such interventions require a better mechanistic understanding of how diet and the microbiome alter cancer promotion.

## Insight into Cancer Incidence

Improved understanding of mechanisms of promotion may also improve our understanding of both clinical and geographical differences in disease incidence. The oncogenic landscape of normal tissues will depend on both the clones that are generated and the clones that are fit enough to survive within the microenvironment, both of which may be altered by environmental exposures. First, exposure to both mutagens and inflammatory agents varies with geography around the world, contributing to disparities in disease incidence. For example, retrospective studies from the People’s Republic of China have found that improved cooking ventilation associated with changing from unvented coal stoves to stoves with chimneys or portable stoves was associated with a reduction in lung cancer incidence (41). The timing and length of exposure to promoters is also an important consideration. In Taiwan, changes in air pollution exposure are beginning to be reflected in altered lung cancer incidence 15 to 20 years later (41). In addition, acute exposure to high levels of traffic-related air pollution during commuting on public transport was associated with an elevated risk of lung cancer compared with automobile use (42). Occupational exposure to asbestos is associated with the development of mesothelioma, but the time between the initial exposure and disease onset is between 10 and 60 years, with a relationship proposed between the total exposed dose and age of onset (43). However, in the case of asbestos, risk of disease does not drop after cessation of exposure, potentially due to the long retention of asbestos particles (44). In contrast, upon cessation of tobacco smoking, it has been proposed that mitotically quiescent cells that have avoided tobacco mutagenesis replenish the lung bronchial epithelium, which contributes to the decreased risk of lung cancer following smoking cessation (45). Recent work in mouse models has also highlighted the possibility of epithelial memory via epigenetic alterations that prime pancreatic cells for later oncogenic transformation (46). This highlights how the latency of cancer following exposure is dependent on tissue-specific biology of tumor initiation. Understanding the relationship between exposure to promoter agents and cancer development will help inform therapeutic windows for intervention.

Second, the magnitude of response to a promoter agent could underlie clinical differences in disease incidence. For example, germline genetic variants are responsible for variation in immune cell levels and hence could alter response to tissue challenge and cancer promotion (47). Another example is in sexual dimorphism in cancer incidence, with many cancers occurring more frequently in males (48). It has been proposed that females are better at mounting and resolving inflammatory responses to stimuli, in part due to estrogen’s ability to dampen inflammatory cytokines such as IL-6 and TNF (48). It is plausible that different immune responses between males and females or people of different genetic ancestry, in response to environmental carcinogens, will alter the likelihood of tumor initiation.

## Opportunities for Intervention

Understanding cancer promotion may offer opportunities to prevent cancer and lower cancer incidence. By improving screening methods for environmental carcinogens that act as promoters, new carcinogens may be identified or substantiate evidence for suspected carcinogens. Understanding the molecular basis for how non-mutagenic carcinogens might promote cancer in at-risk populations might offer opportunities for molecular cancer prevention programs and targeted intervention Cancer prevention trials face a number of hurdles. They require a large clinical cohort to be powered to measure benefit, extended study periods to measure cancer incidence or mortality, a large therapeutic window, and a favorable risk–benefit ratio for an individual without a cancer diagnosis (49). A deep molecular understanding of the mechanisms of early tumorigenesis is fundamental to identifying targets for therapeutic intervention and high-risk populations who will most benefit. As a proof of principle, the canakinumab anti-inflammatory thrombosis outcome study (CANTOS), a double-blind, placebo-controlled phase 3 trial in 10,061 patients with history of myocardial infarction with atherosclerosis were administered with canakinumab, a human mAb targeting IL-1β, with a primary endpoint of recurrent cardiovascular events. Interestingly, whilst not a pre-specified endpoint, a significant, dose-dependent decrease in the incidence of new lung cancer was observed (50). The highest-dose group had a relative hazard reduction of 67% for total lung cancer and 77% for fatal lung cancer. This hypothesis generating observation supports the inflammatory promotion mechanism for lung cancer and could suggest that IL-1β is a molecular cancer prevention target. However, systemic treatment with canakinumab led to increased fatal infection and sepsis, highlighting the difficulties of systemic immune modulation in healthy individuals (51). Follow-up phase III trials of canakinumab in advanced lung cancer have been negative, suggesting tumor promotion and tumor maintenance are distinct biological processes, emphasizing the need to study early tumor biology (51).

NOTCH1 mutations are highly selected for and take over the majority of the human esophagus by middle age but, by contrast, are comparatively rare in esophageal cancers, suggesting they drive clonal selection but hamper carcinogenesis. Recent research formally tested the role of NOTCH1 in mouse models of esophageal cancer. Using the chemical carcinogen diethylnitrosamine, it was found that tumors were significantly smaller in both Notch1 knockout mice and animals treated with a NOTCH1 function–blocking antibody (52). Loss of functional Notch1 had minimal impact on the normal esophagus, suggesting NOTCH1 may be a potential target to reduce the growth of premalignant esophageal tumors.

**Figure 1:**
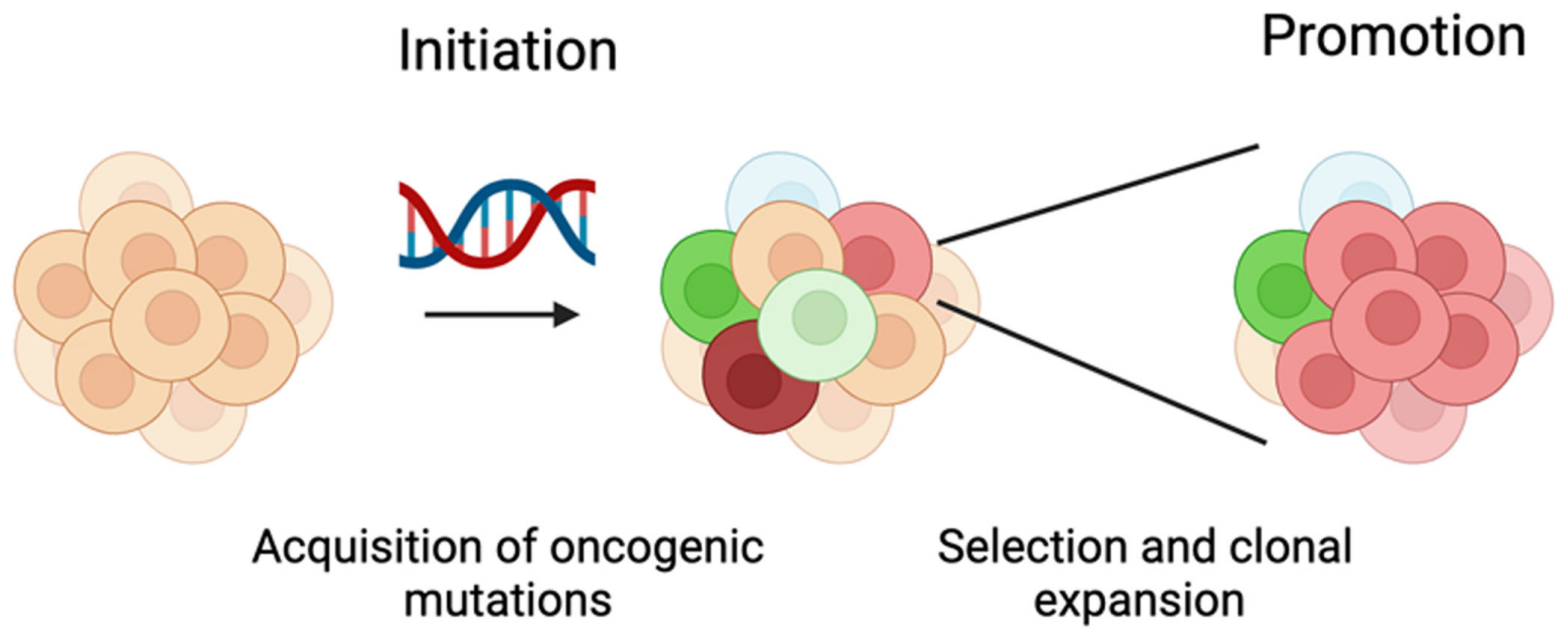
Model of tumor promotion. Over time, cells acquire mutations, some of which will be oncogenic drivers. Altered selection pressures can awaken mutant cells, triggering the earliest stages of tumorigenesis. In the absence of the promoter step, cells are restrained from clonally expanding. (Created with BioRender.com.)

In the colon, stem cells at the base of the crypt compete with neighboring cells. It was recently explored how adenomatous polyposis coli (APC) mutant cells gain a selective advantage and take over the crypt. APC mutant cells upregulate the secreted Wnt deacylase Notum which actively inhibits Wnt signaling in neighboring stem cells (53). This drives neighboring cell differentiation and allows APC mutant cells to expand. Notum inhibitors are currently in development for osteoporosis and neurodegenerative disorders but may also be of use in preventing the earliest stages of colorectal cancer. Conversely, it has also been demonstrated that boosting the fitness of neighboring, wild-type intestinal stem cells by treatment with lithium chloride prevents mutant cells taking over and adenoma formation in mouse models (54). Lithium chloride activates Wnt signaling downstream of secreted Wnt agonists by inhibition of GSK3β. Understanding cell–cell interactions and competition at the earliest stages of cancer can provide novel targets to inhibit mutant cell expansion (Fig. 1).

Inflammatory mediators such as PGE2 are elevated in multiple cancer types due to overexpression of COX-1 and -2. NSAID inhibit both COX-1 and -2, with epidemiological data suggesting potential cancer-preventative effects in colorectal and other gastrointestinal cancers, although their use may be associated with increased risk of some cancers such as lung and bladder (55, 56). Genetic deletion of COX or PGE2 inhibits cancer in subcutaneous tumor models, and pharmacological inhibition using celecoxib was found to be a potent cancer-preventative measure in a mouse intestinal tumor model driven by mutant APC (57, 58). COX ablation leads to an immune shift toward anti-cancer pathways, promoting type I IFN–dependent innate immune cell activation and T-cell-mediated tumor elimination (58). Sequencing of precancerous Barrett’s esophagus also found that treatment with NSAIDs reduces the accumulation of genomic alterations (59). Using epidemiological studies to inform biological research can provide insight into cancer prevention mechanisms.

## Conclusion

**Figure 2:**
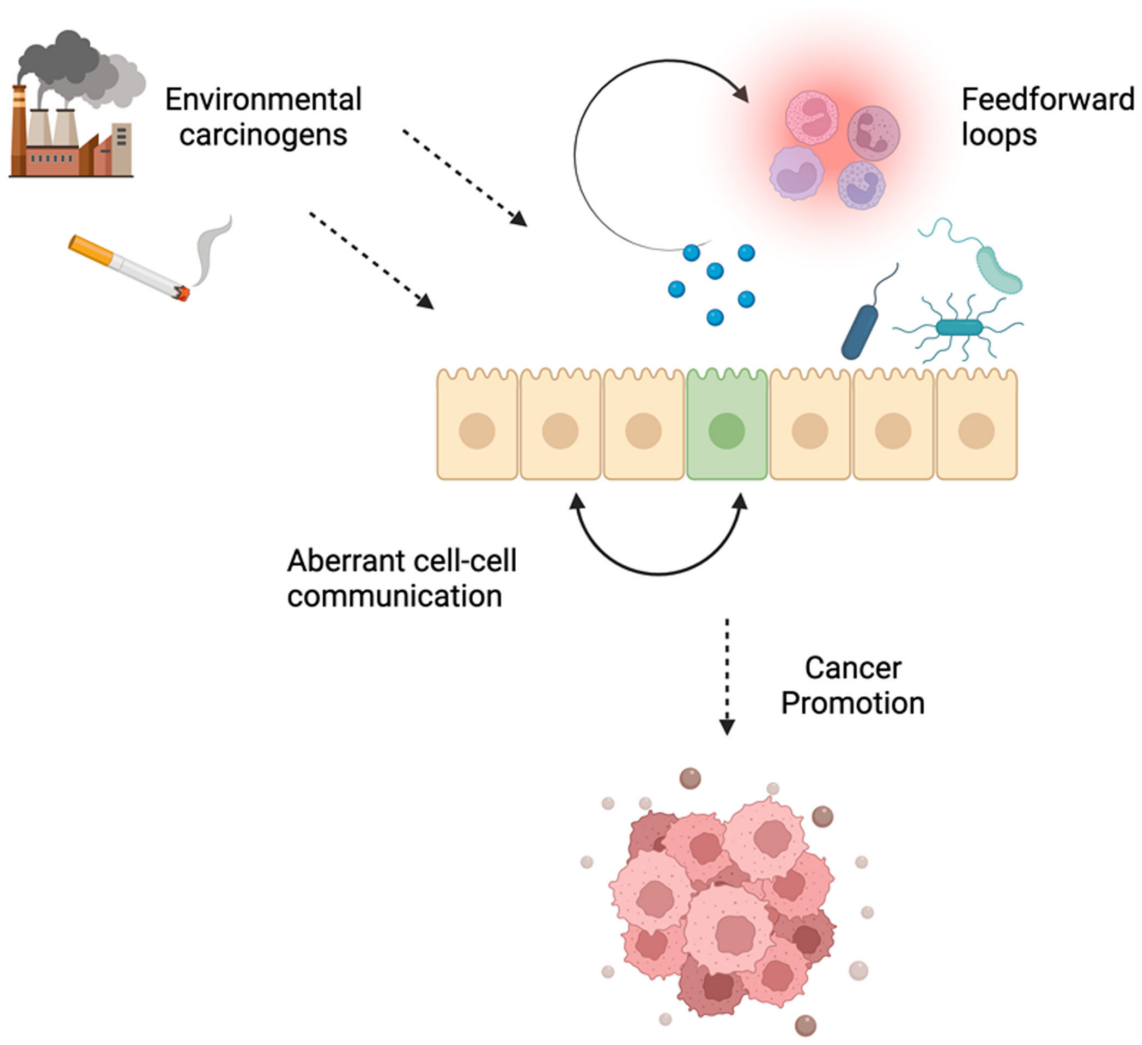
Targeting tumor promotion. Understanding the biological mechanism by which tumor promotion occurs will reveal opportunities for molecular cancer prevention. There is evidence that carcinogens can initiate feedforward loops with the inflammatory microenvironment to fuel pancreatic cancer or drive cytokine production in the lung to promote disease. Mechanistic insight into how mutant stem cells take over the intestine has identified therapies which inhibit mutant cell expansion. (Created with BioRender.com.)

In summary, there is growing evidence that after the acquisition of oncogenic driver mutations, a rate-limiting step for cancer development is the promotion of cancer in some tumor types. Cancer promotion can occur via intrinsic alterations not only in the cell of origin, such as acquisition of progenitor-like phenotypes and epigenetic alterations, but also in the wider microenvironment, such as perturbations to epithelial tissues, including aberrant cell competition, biased daughter cell fate, and immune escape mediated by inflammation and abnormal cell–cell communication (Fig. 2). Understanding the mechanisms of tumor promotion may reveal insight into clinical differences in disease incidence and the rise of early-onset cancer. Importantly, a better understanding of the molecular origins of cancer will identify opportunities for early intervention to prevent cancer.

## Significance

Recent data have improved our understanding of how carcinogens cause cancer, which may reveal novel opportunities for molecular cancer prevention.

## References

[1] Martincorena I, Roshan A, Gerstung M, Ellis P, Van Loo P, McLaren S (2015). Tumor evolution. High burden and pervasive positive selection of somatic mutations in normal human skin. Science.

[2] Muiños F, Martínez-Jiménez F, Pich O, Gonzalez-Perez A, Lopez-Bigas N (2021). In silico saturation mutagenesis of cancer genes. Nature.

[3] Riva L, Pandiri AR, Li YR, Droop A, Hewinson J, Quail MA (2020). The mutational signature profile of known and suspected human carcinogens in mice. Nat Genet.

[4] Moody S, Senkin S, Islam SMA, Wang J, Nasrollahzadeh D, Cortez Cardoso Penha R (2021). Mutational signatures in esophageal squamous cell carcinoma from eight countries with varying incidence. Nat Genet.

[5] Hill W, Lim EL, Weeden CE, Lee C, Augustine M, Chen K (2023). Lung adenocarcinoma promotion by air pollutants. Nature.

[6] Takahashi H, Ogata H, Nishigaki R, Broide DH, Karin M (2010). Tobacco smoke promotes lung tumorigenesis by triggering IKKβ- and JNK1-dependent inflammation. Cancer Cell.

[7] Brennan P, Davey-Smith G (2022). Identifying novel causes of cancers to enhance cancer prevention: new strategies are needed. J Natl Cancer Inst.

[8] Balmain A (2020). The critical roles of somatic mutations and environmental tumor-promoting agents in cancer risk. Nat Genet.

[9] Weeden CE, Hill W, Lim EL, Grönroos E, Swanton C (2023). Impact of risk factors on early cancer evolution. Cell.

[10] Voytek P, Anver M, Thorslund T, Conley J, Anderson E (1990). Mechanisms of asbestos carcinogenicity. J Am Coll Toxicol.

[11] Guerra C, Schuhmacher AJ, Cañamero M, Grippo PJ, Verdaguer L, Pérez-Gallego L (2007). Chronic pancreatitis is essential for induction of pancreatic ductal adenocarcinoma by K-Ras oncogenes in adult mice. Cancer Cell.

[12] Philip B, Roland CL, Daniluk J, Liu Y, Chatterjee D, Gomez SB (2013). A high-fat diet activates oncogenic Kras and COX2 to induce development of pancreatic ductal adenocarcinoma in mice. Gastroenterology.

[13] Caronni N, La Terza F, Vittoria FM, Barbiera G, Mezzanzanica L, Cuzzola V (2023). IL-1β+ macrophages fuel pathogenic inflammation in pancreatic cancer. Nature.

[14] Alonso-Curbelo D, Ho Y-J, Burdziak C, Maag JLV, Morris JP, Chandwani R (2021). A gene-environment-induced epigenetic program initiates tumorigenesis. Nature.

[15] Carpenter ES, Elhossiny AM, Kadiyala P, Li J, McGue J, Griffith BD (2023). Analysis of donor pancreata defines the transcriptomic signature and microenvironment of early neoplastic lesions. Cancer Discov.

[16] Pilley S, Rodriguez TA, Vousden KH (2021). Mutant p53 in cell-cell interactions. Genes Dev.

[17] Hamarsheh S, Groß O, Brummer T, Zeiser R (2020). Immune modulatory effects of oncogenic KRAS in cancer. Nat Commun.

[18] Qin X, Cardoso Rodriguez F, Sufi J, Vlckova P, Claus J, Tape CJ (2023). An oncogenic phenoscape of colonic stem cell polarization. Cell.

[19] Kon S, Ishibashi K, Katoh H, Kitamoto S, Shirai T, Tanaka S (2017). Cell competition with normal epithelial cells promotes apical extrusion of transformed cells through metabolic changes. Nat Cell Biol.

[20] Hill W, Zaragkoulias A, Salvador-Barbero B, Parfitt GJ, Alatsatianos M, Padilha A (2021). EPHA2-dependent outcompetition of KRASG12D mutant cells by wild-type neighbors in the adult pancreas. Curr Biol.

[21] Alcolea MP, Greulich P, Wabik A, Frede J, Simons BD, Jones PH (2014). Differentiation imbalance in single oesophageal progenitor cells causes clonal immortalization and field change. Nat Cell Biol.

[22] Colom B, Alcolea MP, Piedrafita G, Hall MWJ, Wabik A, Dentro SC (2020). Spatial competition shapes the dynamic mutational landscape of normal esophageal epithelium. Nat Genet.

[23] Porazinski S, de Navascués J, Yako Y, Hill W, Jones MR, Maddison R (2016). EphA2 drives the segregation of ras-transformed epithelial cells from normal neighbors. Curr Biol.

[24] Kon S, Fujita Y (2021). Cell competition-induced apical elimination of transformed cells., EDAC, orchestrates the cellular homeostasis. Dev Biol.

[25] Sasaki A, Nagatake T, Egami R, Gu G, Takigawa I, Ikeda W (2018). Obesity suppresses cell-competition-mediated apical elimination of RasV12-transformed cells from epithelial tissues. Cell Rep.

[26] Sato N, Yako Y, Maruyama T, Ishikawa S, Kuromiya K, Tokuoka SM (2020). The COX-2/PGE2 pathway suppresses apical elimination of RasV12-transformed cells from epithelia. Commun Biol.

[27] Mesa KR, Kawaguchi K, Cockburn K, Gonzalez D, Boucher J, Xin T (2018). Homeostatic epidermal stem cell self-renewal is driven by local differentiation. Cell Stem Cell.

[28] Liu N, Matsumura H, Kato T, Ichinose S, Takada A, Namiki T (2019). Stem cell competition orchestrates skin homeostasis and ageing. Nature.

[29] Zhu L, Finkelstein D, Gao C, Shi L, Wang Y, López-Terrada D (2016). Multi-organ mapping of cancer risk. Cell.

[30] Celebi Sözener Z, Cevhertas L, Nadeau K, Akdis M, Akdis CA (2020). Environmental factors in epithelial barrier dysfunction. J Allergy Clin Immunol.

[31] Thong T, Forté CA, Hill EM, Colacino JA (2019). Environmental exposures, stem cells, and cancer. Pharmacol Ther.

[32] Xie C, Zhu J, Wang X, Chen J, Geng S, Wu J (2019). Tobacco smoke induced hepatic cancer stem cell-like properties through IL-33/p38 pathway. J Exp Clin Cancer Res.

[33] Wang J, Chen J, Jiang Y, Shi Y, Zhu J, Xie C (2018). Wnt/β-catenin modulates chronic tobacco smoke exposure-induced acquisition of pulmonary cancer stem cell properties and diallyl trisulfide intervention. Toxicol Lett.

[34] Weeden CE, Chen Y, Ma SB, Hu Y, Ramm G, Sutherland KD (2017). Lung basal stem cells rapidly repair DNA damage using the error-prone nonhomologous end-joining pathway. PLoS Biol.

[35] Vaz M, Hwang SY, Kagiampakis I, Phallen J, Patil A, O’Hagan HM (2017). Chronic cigarette smoke-induced epigenomic changes precede sensitization of bronchial epithelial cells to single step transformation by KRAS mutations. Cancer Cell.

[36] Chakravarti D, LaBella KA, DePinho RA (2021). Telomeres: history, health, and hallmarks of aging. Cell.

[37] Chakravarti D, Hu B, Mao X, Rashid A, Li J, Li J (2020). Telomere dysfunction activates YAP1 to drive tissue inflammation. Nat Commun.

[38] El Tekle G, Garrett WS (2023). Bacteria in cancer initiation, promotion and progression. Nat Rev Cancer.

[39] Zavros Y, Merchant JL (2022). The immune microenvironment in gastric adenocarcinoma. Nat Rev Gastroenterol Hepatol.

[40] Alam A, Levanduski E, Denz P, Villavicencio HS, Bhatta M, Alhorebi L (2022). Fungal mycobiome drives IL-33 secretion and type 2 immunity in pancreatic cancer. Cancer Cell.

[41] Berg CD, Schiller JH, Boffetta P, Cai J, Connolly C, Kerpel-Fronius A (2023). Air pollution and lung cancer: a review by international association for the study of lung cancer early detection and screening committee. J Thorac Oncol.

[42] Wong JYY, Jones RR, Breeze C, Blechter B, Rothman N, Hu W (2021). Commute patterns, residential traffic-related air pollution, and lung cancer risk in the prospective UK Biobank cohort study. Environ Int.

[43] Dragani TA, Colombo F, Pavlisko EN, Roggli VL (2018). Malignant mesothelioma diagnosed at a younger age is associated with heavier asbestos exposure. Carcinogenesis.

[44] Boffetta P, Donato F, Pira E, Luu HN, La Vecchia C (2019). Risk of mesothelioma after cessation of asbestos exposure: a systematic review and meta-regression. Int Arch Occup Environ Health.

[45] Yoshida K, Gowers KHC, Lee-Six H, Chandrasekharan DP, Coorens T, Maughan EF (2020). Tobacco smoking and somatic mutations in human bronchial epithelium. Nature.

[46] Del Poggetto E, Ho I-L, Balestrieri C, Yen E-Y, Zhang S, Citron F (2021). Epithelial memory of inflammation limits tissue damage while promoting pancreatic tumorigenesis. Science.

[47] Orrù V, Steri M, Sole G, Sidore C, Virdis F, Dei M (2013). Genetic variants regulating immune cell levels in health and disease. Cell.

[48] Haupt S, Caramia F, Klein SL, Rubin JB, Haupt Y (2021). Sex disparities matter in cancer development and therapy. Nat Rev Cancer.

[49] Maresso KC, Tsai KY, Brown PH, Szabo E, Lippman S, Hawk ET (2015). Molecular cancer prevention: current status and future directions. CA Cancer J Clin.

[50] Ridker PM, MacFadyen JG, Thuren T, Everett BM, Libby P, Glynn RJ (2017). Effect of interleukin-1β inhibition with canakinumab on incident lung cancer in patients with atherosclerosis: exploratory results from a randomised, double-blind, placebo-controlled trial. The Lancet.

[51] Lythgoe MP, Prasad V (2022). Repositioning canakinumab for non-small cell lung cancer—important lessons for drug repurposing in oncology. Br J Cancer.

[52] Abby E, Dentro SC, Hall MWJ, Fowler JC, Ong SH, Sood R (2023). Notch1 mutations drive clonal expansion in normal esophageal epithelium but impair tumor growth. Nat Genet.

[53] Flanagan DJ, Pentinmikko N, Luopajärvi K, Willis NJ, Gilroy K, Raven AP (2021). NOTUM from Apc-mutant cells biases clonal competition to initiate cancer. Nature.

[54] van Neerven SM, de Groot NE, Nijman LE, Scicluna BP, van Driel MS, Lecca MC (2021). Apc-mutant cells act as supercompetitors in intestinal tumour initiation. Nature.

[55] Skriver C, Maltesen T, Dehlendorff C, Skovlund CW, Schmidt M, Sørensen HT (2023). Long-term aspirin use and cancer risk: a 20-year cohort study. J Natl Cancer Inst.

[56] Bosetti C, Santucci C, Gallus S, Martinetti M, La Vecchia C (2020). Aspirin and the risk of colorectal and other digestive tract cancers: an updated meta-analysis through 2019. Ann Oncol.

[57] Jacoby RF, Seibert K, Cole CE, Kelloff G, Lubet RA (2000). The cyclooxygenase-2 inhibitor celecoxib is a potent preventive and therapeutic agent in the min mouse model of adenomatous polyposis. Cancer Res.

[58] Zelenay S, van der Veen AG, Böttcher JP, Snelgrove KJ, Rogers N, Acton SE (2015). Cyclooxygenase-dependent tumor growth through evasion of immunity. Cell.

[59] Kostadinov RL, Kuhner MK, Li X, Sanchez CA, Galipeau PC, Paulson TG (2013). NSAIDs modulate clonal evolution in Barrett’s esophagus. Public Libr Sci.

